# Optimized Method Based on Subspace Merging for Spectral Reflectance Recovery

**DOI:** 10.3390/s23063056

**Published:** 2023-03-12

**Authors:** Yifan Xiong, Guangyuan Wu, Xiaozhou Li

**Affiliations:** 1 Faculty of Light Industry, Qilu University of Technology (Shandong Academy of Sciences), Jinan 250353, China; 2 State Key Laboratory of Biobased Material and Green Papermaking, Qilu University of Technology (Shandong Academy of Sciences), Jinan 250353, China

**Keywords:** spectral recovery, camera responses, representative samples, subspace merging

## Abstract

The similarity between samples is an important factor for spectral reflectance recovery. The current way of selecting samples after dividing dataset does not take subspace merging into account. An optimized method based on subspace merging for spectral recovery is proposed from single RGB trichromatic values in this paper. Each training sample is equivalent to a separate subspace, and the subspaces are merged according to the Euclidean distance. The merged center point for each subspace is obtained through many iterations, and subspace tracking is used to determine the subspace where each testing sample is located for spectral recovery. After obtaining the center points, these center points are not the actual points in the training samples. The nearest distance principle is used to replace the center points with the point in the training samples, which is the process of representative sample selection. Finally, these representative samples are used for spectral recovery. The effectiveness of the proposed method is tested by comparing it with the existing methods under different illuminants and cameras. Through the experiments, the results show that the proposed method not only shows good results in terms of spectral and colorimetric accuracy, but also in the selection representative samples.

## 1. Introduction

Spectral reflectance is an inherent nature of matter itself, which can be regarded as the ‘fingerprint’ of an object [1]. Due to its unique properties, it can be used to characterize the color of materials and the surface properties of materials in art [2,3,4], remote sensing [5,6], medicine [7,8,9], textiles [7,10] and so on [11,12]. In real life, it is difficult to directly obtain the surface spectral reflectance of an object; researchers mostly obtain the surface spectral reflectance by indirectly obtaining the response value of a digital camera [9]. The response values are usually obtained by using digital devices, which saves time and effort. Therefore, there are many kinds of spectral recovery methods, such as the most common pseudo-inverse method [13,14], principal component analysis method [15], compressive sensing [16,17], Wiener estimation method [8,18], and so on [19,20,21,22,23,24,25]. In the above description, the pseudo-inverse method is more common in the process of spectral recovery.

The similarity of samples is one of the methods to improve the accuracy of spectral recovery. The selection of samples has two major directions: the first is extensive, which mainly selects several fixed representative samples from significant data. Hardeberg [26] proposed a method based on the minimum condition number. Mohammadi [27] proposed a method based on hierarchical clustering. Cheung [28] proposed four optimal sample selection rules based on different criteria between subsequent samples and representative sample subsets. Shen [29] proposed a representative sample selection method based on representative vectors and virtual imaging. Liang [30] proposed representative samples based on the minimum of the defined simulated spectral recovery error. The second is the partition acquisition: the training samples are divided into subspaces, and the test sample is used to select the training sample. The methods proposed above can be simply defined as dynamic partitioning and stationary partitioning. For example, Zhang [31] divided Munsell data into 11 regions, which is stationary partitioning. Zhang [32] selected testing samples by distance, and Liang [1] used the nearest training sample as the training subspace, which are examples of dynamic partitioning. Xiong [33] used dynamic partitional clustering to recover the spectral reflectance from camera response values.

Although the above methods all consider the selection of training samples and reduce the redundancy of data, they do not consider that the sample subsets can also be merged and optimized.

Their limitation is that they only use the differences between the samples, which is often referred to as the competition among the samples, and they do not consider the merging of the sample. Additionally, these methods do not consider the problem of data redundancy. This will increase the amount of computation and increase the cost. Our method takes both cases into account and conducts separate experiments.

In this paper, a novel spectral recovery method based on the merging of subspace is proposed. In this method, each testing sample is taken as an independent sample subspace, which is merged according to the distance between sample subspaces to obtain the final partition information. Similarly, we use the nearest training sample to substitute virtual points as the representative samples to recover the spectral reflectance. The approach can be divided into two parts:

1. Firstly, the training samples are divided into independent classes. We use the subspace concept to treat each class as a subspace, and the subspaces are merged according to the set distance. Secondly, the distance of the first subspace is calculated from the second subspace. If the distance is less than the set distance, then the average between the first subspace and the second subspace is calculated as the first subspace. If the distance is greater than the set distance, then the relationship between the first point and the third point is calculated, which will reduce the number of subspaces and yield partition information. Thirdly, the final merged center points are obtained through many iterations, which are used to determine the subspace. Finally, subspace tracking is used to determine the subspace where each testing sample is located for spectral recovery.
2. After obtaining the center points, these center points are not actual points in the training samples. The nearest distance principle is used to replace the center points with the points in training samples, which is the process of representative sample selection. Finally, these representative samples are used for spectral recovery.

### Mathematic Background and Method

In this section, the imaging function of digital devices is introduced. When the light source distribution function I(λ), camera sensitivity function q(λ) and spectral reflectance r(λ) are determined, the integral process can be expressed as:

(1) $$T=\int_{min}^{max}I\left(\lambda \right)q\left(\lambda \right)r\left(\lambda \right)d\lambda +\gamma ,$$

where *T =* [*R*, *G*, *B*] *^+^* is the RGB response values; + means transpose; min and max represent the visible wavelength range (400 nm–700 nm); the system noise is normally represented by γ, which is omitted in this study [34]; Equation (1) can be further simplified in matrix vector form.

(2) T=MR,

where *M* represents the integral matrix of the light source distribution function and the camera sensitivity function; *R* denotes the spectral reflectance. The inverse solution yields the spectral reflectance recovery to be obtained by Equation (3)

(3) R=QT,

where *Q* is the transpose matrix, and we can easily obtain the spectral reflectance by knowing *Q*.

## 2. The Proposed Method

**Figure 1:**
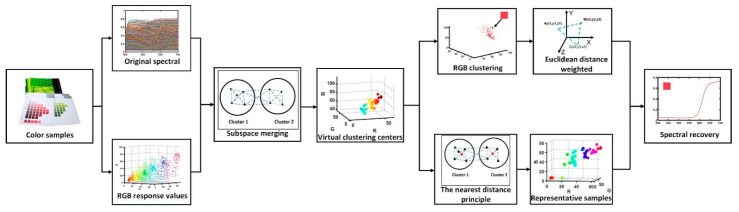
Schematic illustration of the proposed method for spectral reflectance recovery.

In this section, we first show the schematic illustration of the proposed method for spectral reflectance recovery in Figure 1, and formulate the spectral reflectance recovery process based on subspace merging.

As can be seen in Figure 1, firstly, the response values and spectral reflectance information of the color samples are obtained. Secondly, after obtaining the corresponding response value information, subspace merging is carried out according to the distance. Finally, after the merging points are obtained, we can divide it into two categories. The first type is directly partitioned for spectral recovery, and the second type is used in the latest training sample data to replace virtual points in order to directly obtain the restored spectral reflectance.

### 2.1. Spectral Reflectance Recovery Based on Subspace Merging

For the response values, the obtained response values will change due to the difference of spectral sensitivity function and light source. So, the obtained raw response values need to be normalized. The process of standardization is shown in Equations (4) and (5)

(4) H=argmax(T),

(5) T=(T/H)×255,

where argmax describes the function to determine the maximum value of response values. When the maximum value is known, the response values are divided by the maximum and multiplied by 255.

Given the training sample ($x_{1}$...$x_{m}$) $\in T_{Train}$, where *m* represents the number of training samples, let $x_{1}$= ($r_{1},$ $g_{1},\;b_{1}$), since each samples represents a subspace, which is based on the Euclidean distance as the standard to start merging. The initial distance *k* is given. The distance is calculated in Equation (6)

(6) $$s_{j}=\sqrt{{\left(r_{j}-r_{i}\right)}^{2}+{\left(g_{j}-g_{i}\right)}^{2}+b_{j}-b_{i}{}^{2}\;},$$

where *j* is the initial value of the *j*th sample; *i* is the remaining sample points; when *k* < $s_{j}$, proceed to the next step; when *k >* $s_{j}$ the two samples are combined and used as the starting samples of the *j*th point to repeat Equation (6).

(7) $$r_{j}=(r_{j}+r_{i})/2\;g_{j}=\left(g_{j}+g_{i}\right)/2\;b_{j}=\left(b_{j}+b_{i}\right)/2,$$

At the end of the process of comparing the distance with *k* and continuously fusing, all the samples are gathered in a new set *J*. The number of points in *J* varies depending on the value of *k*. We set the number of *J* to *n*. The partition information of the subspaces *C* is found by the division of clustering centers.

(8) $$W\;\left(C,J\right)=min_{C}\sum_{J=1}^{a}\sum_{x_{i}\in C_{a}}\|x_{i}-J_{a}{\|}_{2}^{2}\;\left(i=1,2,\ldots m,a=1,2,\ldots n\right),$$

where the W (C,J) defines the partitioning of the samples and the generation of new clustering centers; $\|\cdot {\|}_{2}^{2}$ represents the Euclidean distance. Let ($p_{1}\ldots p_{f}$)$\in T_{Test}$ be the testing samples. When the testing samples are input into Equation (8), the partition of the training sample set of the test sample is determined.

After the training sample subspace of the testing sample is selected, the distance information between the testing sample and the training sample is used to calculate the weight of the training sample.

(9) $$w_{i}=\|T_{test}-C_{a}{\|}_{2}^{2},$$

where $C_{a}$ represents the corresponding training sample partition of the testing sample. Since the distance is far away, the weight is small. Therefore, its reciprocal is used as the new weighted function. The new weighted function can be expressed as:

(10) $$w_{i}=\frac{1}{w_{i}+\epsilon },$$

Just to make sure the denominator does not equal zero, ϵ = 0.001.

(11) $$W={\left[\begin{array}{cccc} w_{1} & 0 & \cdots & 0\\ 0 & w_{2} & 0 & 0\\ \vdots & 0 & \ddots & \vdots \\ 0 & 0 & \cdots & w_{i} \end{array}\right]}_{i\times i},$$

The spectral recovery function can be expressed as:

(12) $$Q=R_{Train\;}W{\left(T_{Train}W\right)}^{-1},$$

(13) $$R=QT_{Test},$$

The superscript ‘−1’ indicates the pseudo-inverse matrix operator; $R_{Train}$ represents the selected optimal local training sample; $T_{Test}$ represents the standardized response values of the testing sample; $T_{Train}$ represents the standardized response values of the training sample; *R* is the corresponding recovered spectral reflectance.

### 2.2. Representative Samples for Spectral Reflectance Recovery

The center point set *J* can be obtained by using Equation (9) in this section, but the obtained center points are virtual points. The nearest distance principle is used to replace the virtual points with the points in the training samples, which is the process of representative sample selection. The proximate similar substitution principle is used in this section.

(14) $$V=\|x_{i}-J_{a}{\|}_{2}^{2}\left(i=1,2,\ldots m,a=1,2,\ldots n\right),$$

where *V* represents the distance. Firstly, the distance between the first point of *J* and the training sample is calculated, replace the first virtual points of *J* by determining the closest training samples. Then, the selected point is removed from the training samples, and the 2nd to the *n*th point are also replaced.

After obtaining the representative samples set *J*, the final recovered equation can be expressed as:

(15) $$Q=R_{train}{\left(T_{train}\right)}^{-1},$$

(16) $$R=QT_{Test},$$

where $R_{train}$ represents the selected optimal local training sample; $T_{train}$ is the response values of training samples. $T_{Test}$ is the response values of testing samples.

## 3. Experiment

The experiment is divided into two parts: partitioning the acquisition to recover spectral reflectance and selecting the representative samples to recover spectral reflectance. The color difference of CIE DE76 under the CIE 1964 standard observation system and CIE A light source is calculated. The root mean square (RMSE) and goodness-of-fit coefficient (GFC) also as a precision standard. These three parameters are selected as the evaluation standard for spectral recovery.

(17) $$\Delta E_{ab}^{*}=\sqrt{{\left(\Delta L^{*}\right)}^{2}+{\left(\Delta a^{*}\right)}^{2}+{\left(\Delta b^{*}\right)}^{2}},$$

(18) $$\mathrm{RMSE}=\sqrt{\frac{1}{m}{\left(R_{test}-R\right)}^{T}\left(R_{test}-R\right)},$$

(19) $$\mathrm{GFC}=\frac{R_{test}{}^{T\;}R}{\|R_{test}{}^{T}\;R_{test}\|\cdot \|RR\|\;\;\;},$$

The calculation method of color difference is introduced in Equation (17), where $\Delta L^{*}$ represents the difference in brightness; $\Delta a^{*}$ represents the difference in redness and greenness; $\Delta b^{*}$ represents the difference in yellowness and blueness. So, color difference represents the colorimetric accuracy. For Equations (18) and (19), where $R_{test}$ represents the recovery spectral reflectance, *R* represents the original spectral reflectance; in this work, m=31. The root of mean square shows the distance between the original and recovered spectral reflectance. The goodness-of-fit coefficient shows the similarity between the original and recovered spectral reflectance.

### 3.1. Simulation Experiment

The 1269 Munsell Matt chips [27], 140 ColorChecker SG [35] and 354 Vrhel spectral datasets [36] are used in the simulation experiment. Firstly, The Munsell Matt chips are used as the training samples. The Munsell Matt chips have 1269 color chips, which are mostly used in spectral recovery, and there are corresponding color blocks for each hue in this training sample. Therefore, the Munsell Matt chips are more convincing as the training sample. Using only one type of color chip undoubtedly affects the universality and effectiveness of the experiment. So, other color chips and Munsell Matt chips are used together to verify the proposed method.

**Figure 2:**
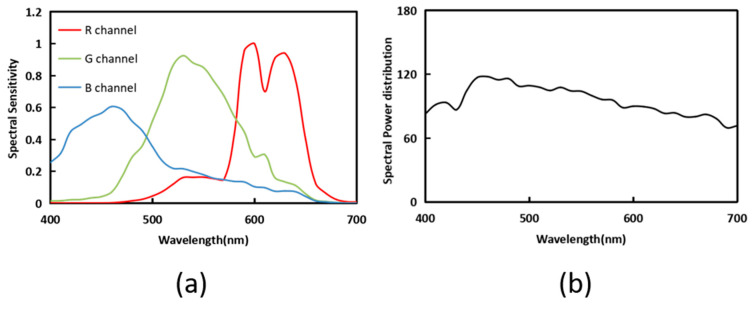
(**a**) Spectral sensitivity functions and (**b**) the spectral power distribution of D65.

The simulated environment is described in this section. The NokiaN900(Nokia Corporation, Espoo, Finland) is selected as the spectral sensitivity function, and the CIE D65 is selected as the light source environment in Figure 2.

**Figure 3:**
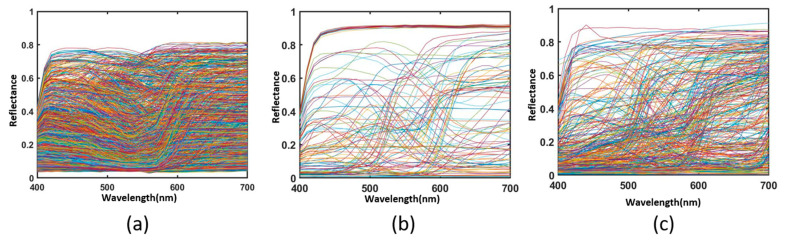
The spectral reflectance of (**a**) 1269 Munsell Matt chips, (**b**) the 140 ColorChecker SG and (**c**) the 354 Vrhel spectral dataset.

All the spectral reflectance data from in the experiment are presented in Figure 3. Figure 3 shows that our experiment involves three kinds of color chips. Figure 3a shows the Munsell Matt chips with 1269 color chips. Figure 3b shows the 140 ColorChecker SG. Figure 3c shows the 354 Vrhel spectral dataset. The spectral reflectance ranges from 400 to 700 at a 10 nm interval.

**Figure 4:**
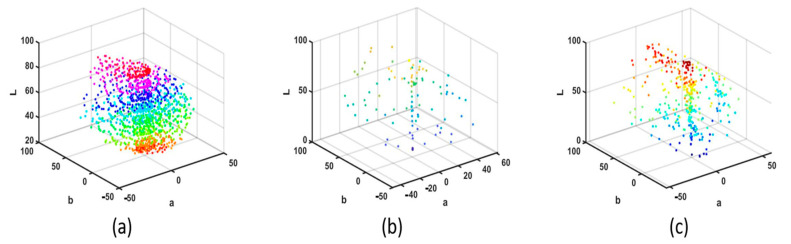
CIE Lab coordinate of (**a**) 1269 Munsell Matt chips, (**b**) the 140 ColorChecker SG and (**c**) the 354 Vrhel spectral dataset.

After analyzing the spectral information data, the response value information is also analyzed. The equipment that obtains the response values greatly depends on the real environment. The camera response value depends a lot on the equipment, which is not a uniform space. In order to facilitate better observation and description of the color, the CIE Lab space with good spatial uniformity is selected as the description background. The full name of Lab is CIELAB, sometimes written as CIE L *a*b*. It is a color pattern developed by the CIE (International Commission on Illumination). Therefore, in Figure 4a, it can be easily seen that the data are distributed uniformly in space, which describes the LAB information of Munsell Matt chips. From Figure 4b,c, it is also easy to see that since the color chips selected are ColorChecker SG and Vrhel spectral dataset, the number is obviously smaller. The LAB is calculated under CIE D65 illuminants.

**Figure 5:**
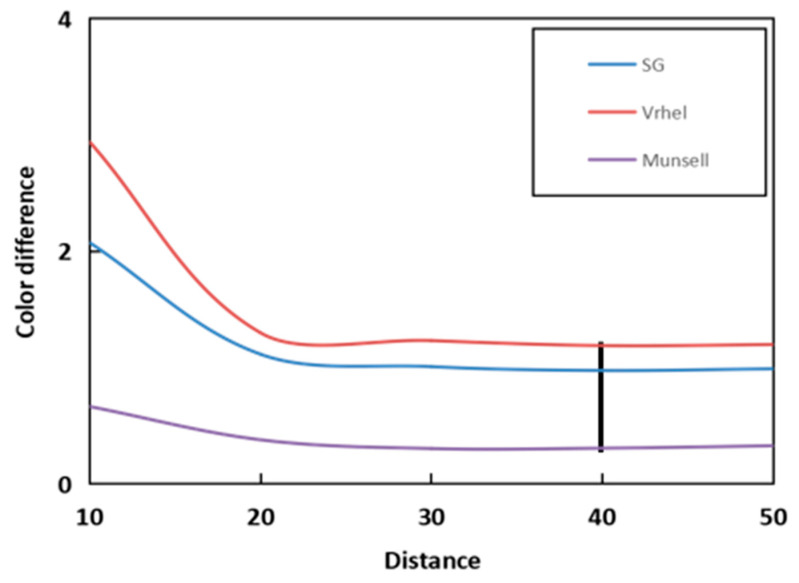
Relationship between the distance and averaged mean color differences.

After the spectral information and response values of the experiment are introduced, the proposed method is tested in the following sections. Distance is used as a parameter in the experiment, and the number of merging iterations between subspaces becomes more and more with the increase in distance. The number of center points is also less and less with the increase in distance, but this does not mean that the center point can directly determine whether the recovery accuracy is good or bad, due to the complex relationship inside. Therefore, the relationship between parameters and accuracy is explored through experiments. In Figure 5, the Munsell Matt chips are used as the training samples. It can be easily seen that both the self-recovery and the recovery of the ColorChecker SG and Vrhel spectral datasets have the best results under the distance of 40. As can be seen from Figure 5, when the Munsell Matt chips are used to recover the other three kinds of data, the results all have the same trend of change. So, the distance is set to 40.

As we can see from Table 1, the proposed method, PI, PCA and Wang’s [37] and Zhang’s [32] methods recover spectral reflectance under the same conditions. The evaluation of the recovery accuracy can be divided into two parts: colorimetric accuracy and spectral accuracy. Firstly, colorimetric analysis is the analysis of color difference. For the color difference analysis in Table 1, it can be seen that the average color difference obtained by either self-recovery or using other samples as training samples using the proposed method is the smallest. The smallest average color difference is self-recovery, which is 0.3063. Secondly, spectral accuracy analysis is the RMSE and GFC. The RMSE and GFC show the same results. The best results are in bold.

**Figure 6:**
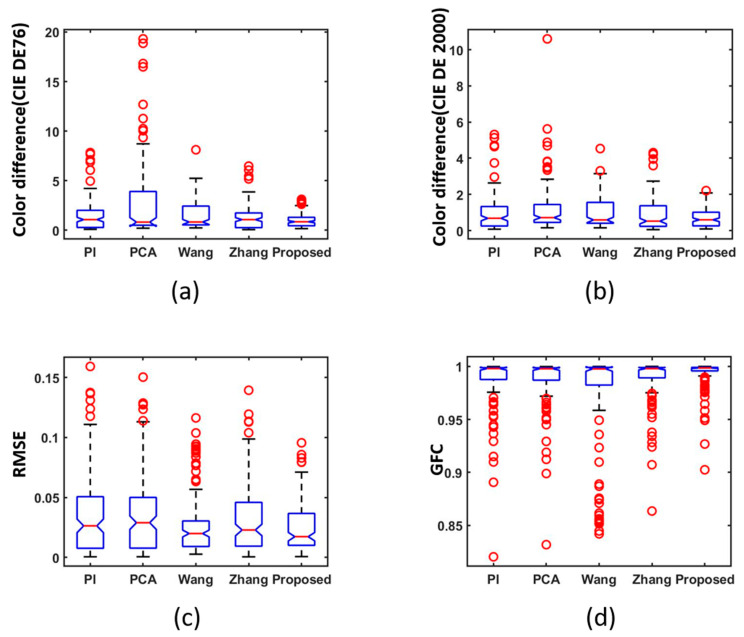
Boxplots of simulated experiment results: (**a**) CIEDE 1976 color difference, (**b**) CIE DE2000 color difference, (**c**) RMSE and (**d**) GFC.

To visualize the recovery accuracy, a boxplot is used in Figure 6. Munsell Matt chips are used as training samples and ColorChecker SG is used as the testing sample. Figure 6a represents CIE DE76 color difference and Figure 6b represents CIE DE2000 color difference. Figure 6c represents RMSE and Figure 6b represents GFC. The more compact the box, the better the precision. It is not difficult to conclude that the proposed method shows better performance. In Figure 6a–c, it can be seen that the distance between the red dots is relatively close, and the accuracy of recovery is more stable than other methods. There are six important data points related to a boxplot: upper edge, lower edge, upper quartile, lower quartile, the median, and outlier. The upper and lower solid black lines represent the upper and lower edge values. The top and bottom of the blue box line indicate the top and bottom quartiles. The red color inside the box indicates the median, and red circles represent outliers.

**Figure 7:**
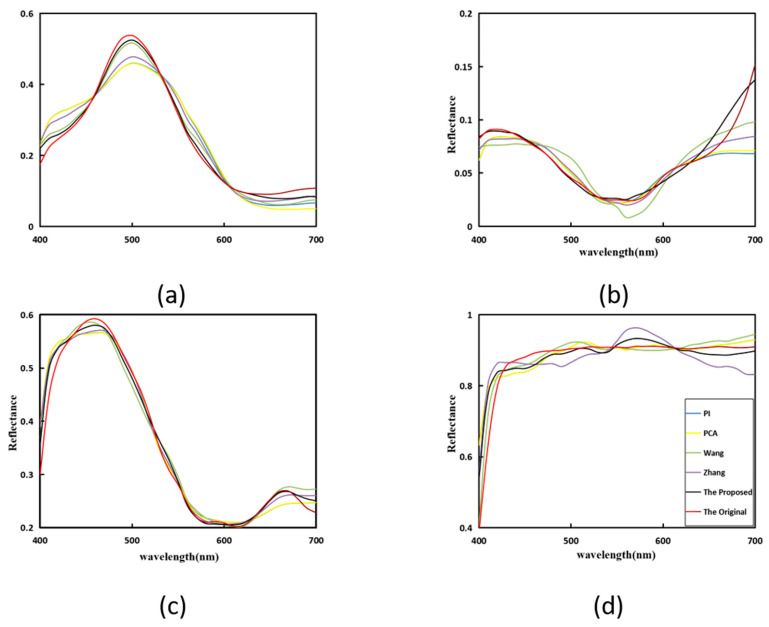
Spectral reflectance recovery results from our proposed and existing methods with four randomly selected samples (Wang [37], Zhang [32]): (**a**) 18, (**b**) 22, (**c**) 92, and (**d**) 40.

In Figure 7, Munsell Matt chips are used as training samples and ColorChecker SG is used as the testing sample. Four random samples are selected for comparison. It can be easily seen that the proposed method is closer to the original sample, so the proposed method shows better performance.

In Figure 7, different colors represent different methods. Correspond to the color of the method in the Figure 7d. After simple verification of the proposed method, in order to show the good performance of the method, which is applied to the spectral images [27], the spectral images ColorChecker and fruitandflowers are used.

**Figure 8:**
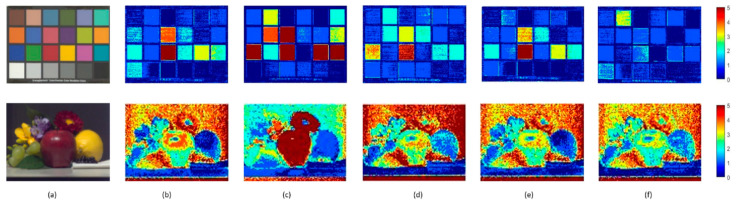
Results comparison of spectral images in different methods using the NokiaN900 as the spectral sensitivity: (**a**) the RGB image, (**b**) PI, (**c**) PCA, (**d**) Wang [37], (**e**) Zhang [32] and (**f**) the proposed.

It can easily be seen in Figure 8 that the results comparison of the spectral images uses the different methods to recover spectral reflectance. Figure 8a represents the original RGB image. Figure 8b–f is called the error map, which calculates the color difference of the spectral reflectance recovered by different methods. More red means a larger color difference, and more blue means a lesser color difference. Therefore, the proposed method shows better performance.

Previous research results show that so many training samples also contain sample redundancy in the field of spectral recovery. It is not necessary to use all samples in the database as training samples. Otherwise, it will cause a heavy workload and inconvenience for sample collection and processing, especially in outdoor applications. Therefore, the optimal selection of representative samples from existing databases has always been an important aspect of spectral recovery.

According to Table 2, using different distances will select the corresponding representative samples. As the distance increases, the accuracy shows a trend of first increasing, then decreasing.

**Figure 9:**
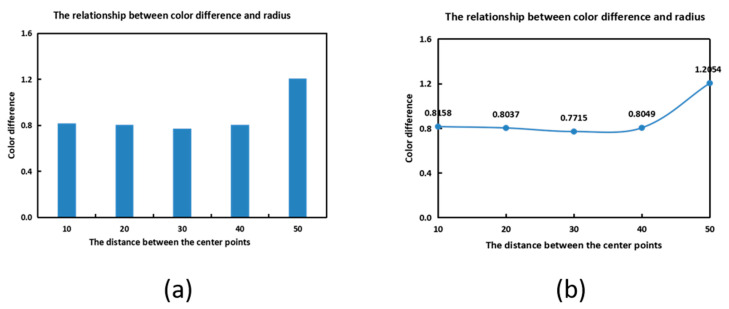
The relationship between mean color difference and distance (**a**) Columnar statistical chart and (**b**) Linear statistical graph.

As can be seen from Figure 9, the results show that there is an extreme value of recovery accuracy, which is very similar to Figure 5. It is also a concave linear curve with a small amplitude, which shows better properties at the distance of 30, and the selected sample is 24. So, we set the distance to 30 to determine the representative samples. The comparison of the proposed method using representative points and some current methods is shown in Table 3.

**Figure 10:**
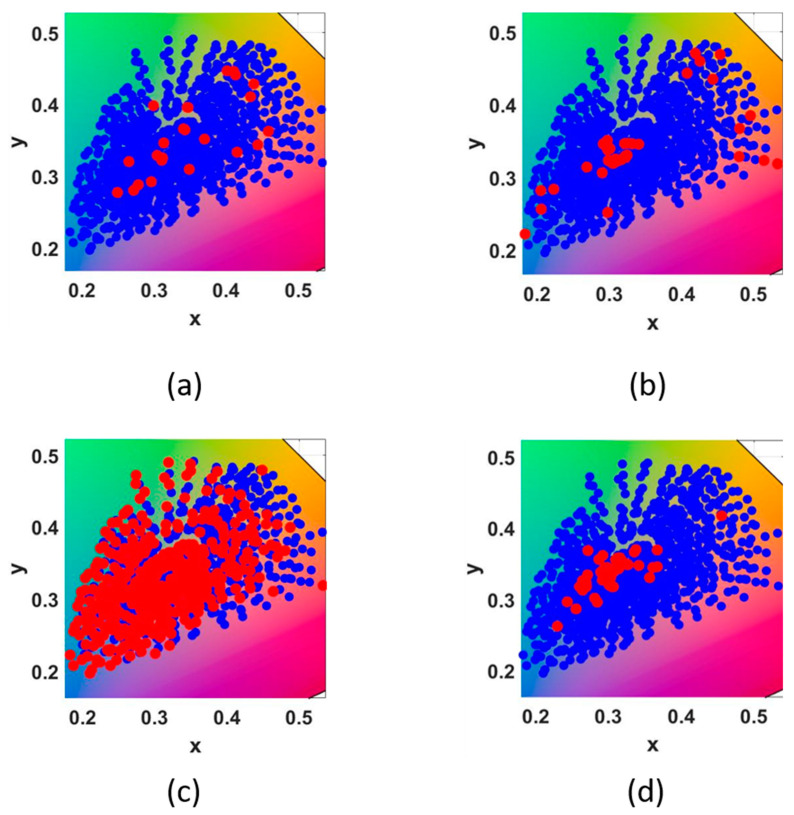
The distribution of training samples and representative points selected by four methods in xyY space, respectively: (**a**) the proposed, (**b**) Cheung [28], (**c**) Hardeberg [26] and (**d**) Liang [30].

**Figure 11:**
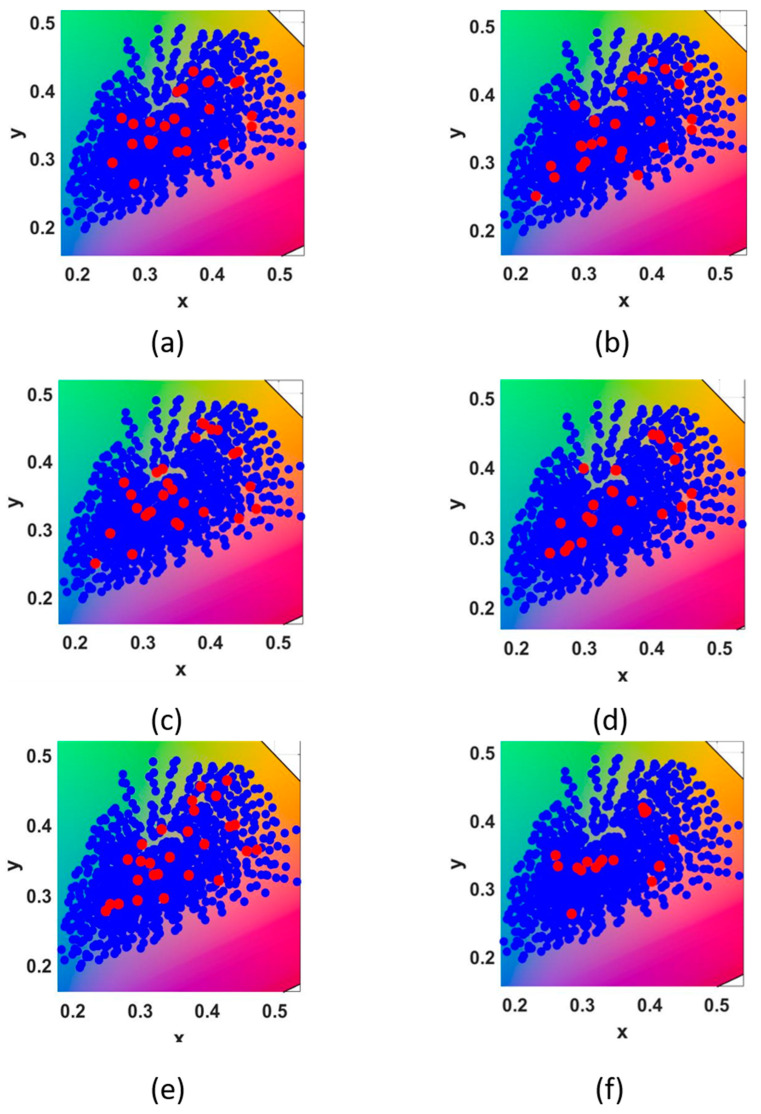
The distribution of training samples and representative samples selected by different illuminants in xyY space, respectively: (**a**) B, (**b**) C, (**c**) D50, (**d**) D65, (**e**) E and (**f**) F2.

After processing selected representative samples, Figure 10 shows the distribution of the training samples and the representative samples selected by several methods in the xyY space. Blue points represent training points and red points represent representative points. Timo Eckhard [38] discussed the sample selection method proposed above, and we select his detection results as the selection samples. Liang’s [30] selected sample used 60 in his article. Figure 10a represents the distribution of selected samples obtained by the proposed method. Figure 10b uses Cheung’s method to calculate the distribution of the selected samples in the training sample. Figure 10c represents Hardburg’s method to calculate the distribution of the selected samples in the training sample. Figure 10d uses Liang’s method to obtain the selected samples. The red dots represent the selected representative points, and the blue dots represent the overall data in Figure 10 and Figure 11.

### 3.2. Recovery for Different Illuminants and Cameras

Considering that different illuminants’ [24,39] and cameras’ spectral sensitivities will affect the proposed method, the Munsell chips are used as training samples, and different illuminants and spectral sensitivity functions are used to verify the effectiveness of the proposed method in Table 4, Table 5, Table 6 and Table 7. Then, the results are recovered according to the ColorChecker SG data testing sample.

Table 4 shows the results of RMSE, GFC and color difference calculated by each comparison method under different illuminants. Five kinds of illuminants are used in Table 4, which are CIE illuminant B, CIE illuminant C, CIE illuminant D50, CIE illuminant D65, CIE illuminant E, and CIE illuminant F2, respectively. It is easy to see that both the mean of color difference and the average value obtained by RMSE and GFC show better performance.

Table 5 shows the results of the whole recovery by selecting representative samples under different illuminants. The proposed method shows better performance in more scenarios.

As can be seen from Figure 11, the distribution of representative samples with the distance of 30 under different illuminants is shown.

The experiments are performed by using the spectral sensitivity of different commercial cameras. The results are general due to different spectral sensitivities. The red, green and blue channels of the digital camera replace the NokiaN900 sensitivity function mentioned above, which is the database of camera sensitivity functions measured by Jiang in 2013 [40].

**Figure 12:**
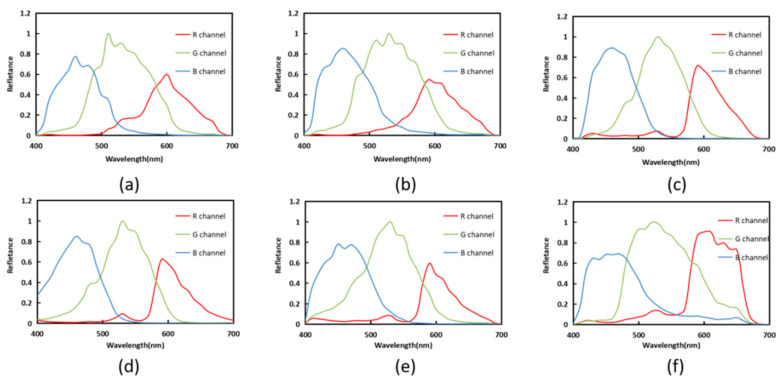
The distribution of spectral sensitivity functions of four selected digital cameras: (**a**) Canon5D Mark II, (**b**) Canon60D, (**c**) Nikon D3, (**d**) Nikon D50, (**e**) PentaK5 and (**f**) Sony Nex5N.

The results in Table 6 are obtained using Munsell Matt chips as training samples and ColorChecker SG data as testing samples. The spectral sensitivity functions in Figure 12 are used as the observer condition. The results are the same as those shown in Table 4, and the proposed method shows better results in terms of mean values.

After the spectral sensitivities are introduced, Table 6 shows the results of several recovery methods using different camera sensitivities. Table 7 shows the results of several recovery methods using different illuminants under Canon5D Mark II spectral sensitivity function after the exploration of the illuminants and spectral sensitivity. Recovery accuracy is demonstrated by using spectral maps.

**Figure 13:**
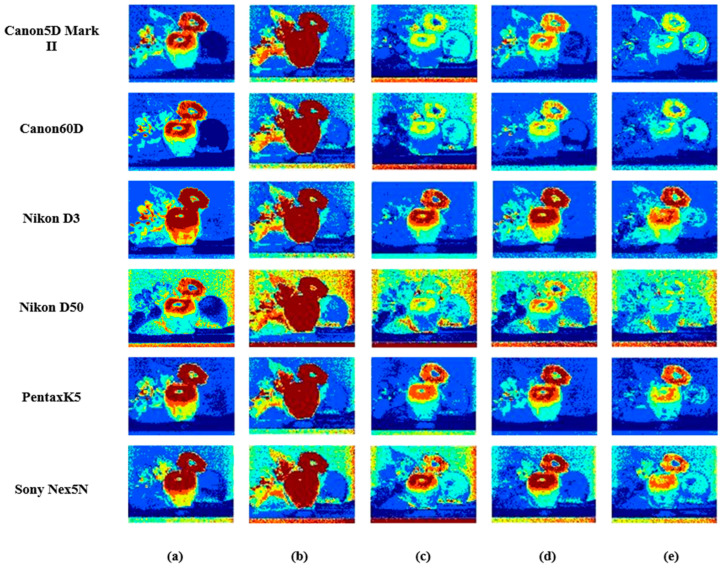
Results comparison of fruit spectral images in different methods using six spectral sensitivity functions as the spectral sensitivity: (**a**) PI, (**b**) PCA, (**c**) Wang [37], (**d**) Zhang [32] and (**e**) the proposed.

**Figure 14:**
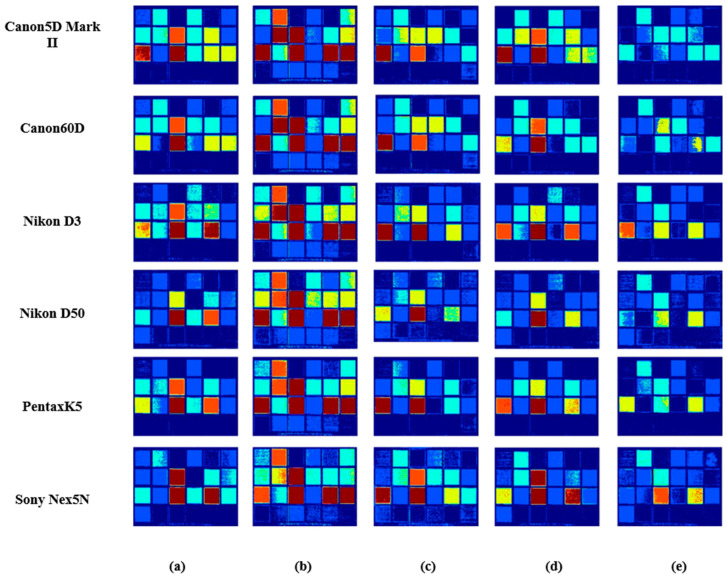
Results comparison of ColorChecker spectral images in different methods using six spectral sensitivity functions as the spectral sensitivity: (**a**) PI, (**b**) PCA, (**c**) Wang [37], (**d**) Zhang [32] and (**e**) the proposed.

Figure 13 and Figure 14 both use Munsell as the training sample to obtain the response value under the Canon5D Mark II and the illuminant CIE D65. Figure 13 shows fruitandflowers as the testing sample and Figure 14 shows ColorChecker as the testing sample. Their error bars are the same as in Figure 5. Figure 13 and Figure 14 show the results obtained with six sensitivity functions using different spectral recovery methods. The range of color from blue to red indicates that the error ranges from small to large. The results show that the proposed method is superior to other methods.

## 4. Discussion and Conclusions

In this study, an optimized method based on subspace merging for spectral reflectance recovery is proposed. The optimal training distance is selected to determine the subspace where the training samples are located, and then the subspace where the testing samples are located is selected according to the subspace tracing for spectral recovery. In this paper, the merged points are also used to determine the representative samples from the large number of training samples. In this experiment, three kinds of samples, six kinds of illuminants and six kinds of camera spectral sensitivity functions are selected. The results show that the best recovery effect is achieved when the Euclidean distance is 40. For the selection of representative points, the recovery effect of the overall sample is better when the Euclidean distance is 30.

The results shows whether Munsell chips recover themselves or recover ColorCheck SG and Vrhel. The best results are obtained when using the proposed Munsell method to recover Munsell, and the color difference is 0.3063. Under the spectral sensitivity function of NokiaN900, the mean color difference of the changed illuminants is still the minimum 0.9546 and the GFC reaches 0.9998, which indicates that both spectral accuracy and colorimetric accuracy have good performance. After changing the spectral sensitivity, a different camera sensitivity function is used. The proposed method’s average color difference is 0.9441 and reaches 0.9999. Canon5D Mark II spectral sensitivity function is used under different illuminants to calculate the error. The average color difference is 0.9982. The average of GFC of 0.9999 is the largest. You can see whether it is sensitive to different camera functions or different illuminants. All the proposed methods show good performance.

In future research, we will conduct more tests on the method in different application fields. In future research, we will try to explore real mineral color chips.

## Figures and Tables

**Table 1:** Results of recovered spectral reflectance for different testing samples using different spectral recovery methods.

			RMSE		GFC		$\Delta E_{ab}$	
Testing Sample		Methods	Mean	Max	Mean	Max	Mean	Max
Munsell		PI	0.0212	0.1387	0.9942	0.9999	0.8142	6.8360
		PCA	0.0218	0.1310	0.9942	0.9999	1.8844	16.9725
		Wang [37]	0.0341	0.1688	0.9844	0.9999	1.5788	5.9162
		Zhang [32]	0.0192	0.1234	0.9950	1.0000	0.7617	5.8412
		Proposed	**0.0103**	**0.1028**	**0.9985**	**1.0000**	**0.3063**	**2.1240**
ColorChecker SG		PI	0.0344	0.1590	0.9883	0.9997	1.4405	7.8373
		PCA	0.0332	0.1503	0.9886	0.9997	2.7231	19.3066
		Wang [37]	0.0267	0.1163	0.9765	0.9998	1.4212	8.1198
		Zhang [32]	0.0312	0.1393	0.9897	0.9999	1.3053	6.4735
		Proposed	**0.0236**	**0.0955**	**0.9932**	**0.9998**	**0.9743**	**3.1183**
Vrhel		PI	0.0383	0.1882	0.9841	0.9997	1.7829	7.7830
		PCA	0.0379	0.1870	0.9843	0.9997	3.2865	20.4141
		Wang [37]	0.0341	**0.1688**	0.9843	0.9999	1.5775	**5.9369**
		Zhang [32]	0.0350	0.1837	0.9851	0.9999	1.5588	6.5949
		Proposed	**0.0316**	0.1706	**0.9866**	**0.9999**	**1.1885**	6.7224

**Table 2:** The relationship between recovery accuracy and distance.

Distance	Selected Samples	Color Difference
10	241	0.8158
20	63	0.8037
30	24	0.7715
40	13	0.8049
50	8	1.2054

**Table 3:** Compares recovery error for Optimal Selected Training Samples in difference methods.

Method	Selected Samples	Mean RMSE	$\Delta E_{ab}$
The proposed	24	0.0214	0.7715
Cheung [28]	35	0.0285	1.2434
Hardeberg [26]	485	0.0230	0.8031
Liang [30]	60	0.0219	0.8099

**Table 4:** Results of recovery spectral reflectance for different illuminants using different spectral recovery methods and NokiaN900 spectral sensitivity function.

			RMSE		GFC		$\Delta E_{ab}$	
Illuminants		Methods	Mean	Max	Mean	Max	Mean	Max
B		PI	0.0331	0.1519	0.9885	0.9997	1.1993	7.6998
		PCA	0.0330	0.1441	0.9888	0.9997	1.7677	13.0748
		Wang [37]	0.0261	0.1063	0.9785	0.9998	1.2564	10.5796
		Zhang [32]	0.0320	0.1292	0.9990	0.9998	3.4029	37.7525
		Proposed	0.0240	0.1129	0.9931	0.9998	0.8474	3.2051
C		PI	0.0334	0.1570	0.9884	0.9997	1.2949	7.2480
		PCA	0.0332	0.1485	0.9887	0.9997	2.4776	17.5926
		Wang [37]	0.0265	0.1131	0.9767	0.9998	11.3332	8.6304
		Zhang [32]	0.0311	0.1368	0.9898	0.9998	2.5704	21.3022
		Proposed	0.0226	0.0938	0.9935	0.9998	0.8105	2.9903
D50		PI	0.0331	0.1535	0.9885	0.9997	1.2897	8.5185
		PCA	0.0330	0.1454	0.9887	0.9997	2.0228	15.0978
		Wang [37]	0.0262	0.1090	0.9783	0.9998	1.2790	10.1201
		Zhang [32]	0.0318	0.1326	0.9899	0.9999	3.3789	40.5513
		Proposed	0.0232	0.1126	0.9932	0.9999	0.8815	3.1797
D65		PI	0.0334	0.1590	0.9883	0.9997	1.4405	7.8373
		PCA	0.0332	0.1503	0.9886	0.9997	2.7231	19.3066
		Wang [37]	0.0267	0.1163	0.9765	0.9998	1.4212	8.1198
		Zhang [32]	0.0312	0.1393	0.9897	0.9999	2.5217	23.0217
		Proposed	0.0236	0.0955	0.9932	0.9998	0.9743	3.1183
E		PI	0.0330	0.1548	0.9884	0.9997	1.6775	8.9601
		PCA	0.0328	0.1467	0.9887	0.9997	2.3032	14.4599
		Wang [37]	0.0263	0.1105	0.9769	0.9998	1.6316	9.1008
		Zhang [32]	0.0300	0.1416	0.9895	0.9998	2.5857	24.9101
		Proposed	0.0237	0.1028	0.9933	0.9998	1.1414	3.9218
F2		PI	0.0356	0.1855	0.9876	0.9997	1.6952	9.1798
		PCA	0.0356	0.1752	0.9877	0.9997	2.3032	14.4599
		Wang [37]	0.0290	0.1388	0.9789	0.9998	1.5170	7.9642
		Zhang [32]	0.0340	0.1670	0.9888	0.9998	4.3534	47.3932
		Proposed	0.0254	0.1110	0.9926	0.9998	1.0724	5.0488
**Average**		PI	0.0336	0.1603	0.9883	0.9997	1.4329	8.2406
		PCA	0.0335	0.1517	0.9885	0.9997	2.2663	15.6653
		Wang [37]	0.0268	0.1157	0.9776	0.9998	3.0731	9.0858
		Zhang [32]	0.0317	0.1411	0.9911	0.9998	3.1355	32.4885
		Proposed	**0.0238**	**0.1048**	**0.9932**	**0.9998**	**0.9546**	**3.5773**

**Table 5:** The spectral recovery color difference of selected representative samples to recover total samples.

	Method			
Illuminants	The Proposed	Cheung [28]	Hardeberg [26]	Liang [30]
B	**0.7059**	1.0274	0.7378	0.7995
C	**0.7380**	1.1504	0.7814	0.7727
D50	0.7699	1.0492	**0.7440**	0.8222
D65	**0.7715**	1.2434	0.8031	0.8099
E	**0.8217**	1.4588	0.9035	0.8827
F2	1.2905	1.3472	1.1153	**1.1046**

**Table 6:** Results of recovery spectral reflectance for different spectral sensitivity functions using different spectral recovery methods.

			RMSE		GFC		$\Delta E_{ab}$	
Spectral Sensitivity		Methods	Mean	Max	Mean	Max	Mean	Max
Canon5D Mark II		PI	0.0352	0.1722	0.9879	0.9997	1.9339	8.7401
		PCA	0.0349	0.1614	0.9882	0.9997	3.5937	30.1884
		Wang [37]	0.0284	0.1171	0.9703	0.9998	3.0478	23.8372
		Zhang [32]	0.0316	0.1533	0.9890	0.9998	1.7473	16.0427
		Proposed	0.0248	0.1135	0.9918	0.9999	1.0844	6.2860
Canon60D		PI	0.0350	0.1778	0.9880	0.9997	1.6355	15.6113
		PCA	0.0347	0.1612	0.9883	0.9997	3.5937	27.5312
		Wang [37]	0.0282	0.1173	0.9704	0.9998	2.8043	20.0648
		Zhang [32]	0.0315	0.1536	0.9891	0.9998	1.5114	13.3564
		Proposed	0.0253	0.1144	0.9918	0.9999	0.9855	5.7372
Nikon D3		PI	0.0354	0.1783	0.9877	0.9997	1.6666	14.9339
		PCA	0.0354	0.1675	0.9880	0.9997	3.9613	35.1863
		Wang [37]	0.0292	0.1270	0.9701	0.9998	2.9183	19.2593
		Zhang [32]	0.0316	0.1601	0.9891	0.9998	1.4146	11.2617
		Proposed	0.0253	0.0988	0.9921	0.9999	0.8508	5.0009
Nikon D50		PI	0.0420	0.1730	0.9882	0.9997	1.4045	9.7738
		PCA	0.0341	0.1632	0.9885	0.9997	4.1587	38.4800
		Wang [37]	0.0283	0.1263	0.9705	0.9998	2.6913	13.1325
		Zhang [32]	0.0309	0.1556	0.9894	0.9998	1.1946	6.9526
		Proposed	0.0249	0.1079	0.9921	0.9999	0.8965	3.0218
PentaxK5		PI	0.0346	0.1697	0.9881	0.9997	1.5506	15.4997
		PCA	0.0345	0.1589	0.9883	0.9997	3.2065	25.2036
		Wang [37]	0.0275	0.1295	0.9778	0.9998	1.3520	11.6586
		Zhang [32]	0.0313	0.1553	0.9891	0.9998	1.4023	13.2577
		Proposed	0.0244	0.1048	0.9922	0.9999	0.7999	5.5646
Sony Nex5N		PI	0.0354	0.1861	0.9876	0.9997	1.6740	12.9780
		PCA	0.0353	0.1749	0.9879	0.9997	3.2214	26.1147
		Wang [37]	0.0291	0.1386	0.9705	0.9998	2.7709	17.0108
		Zhang [32]	0.0326	0.1725	0.9886	0.9998	1.5564	10.7624
		Proposed	0.0253	0.1083	0.9914	0.9999	1.0473	4.9687
**Average**		PI	0.0363	0.1762	0.9879	0.9997	1.6442	12.9228
		PCA	0.03482	0.1645	0.9882	0.9997	3.6226	30.4507
		Wang [37]	0.02845	0.1260	0.9716	0.9998	2.5974	17.4939
		Zhang [32]	0.0316	0.1584	0.9891	0.9998	1.4711	11.9389
		Proposed	**0.0250**	**0.1080**	**0.9919**	**0.9999**	**0.9441**	**5.0965**

**Table 7:** Results of recovery spectral reflectance for different illuminants using different spectral recovery methods and Canon5D Mark II spectral sensitivity function.

			RMSE		GFC		$\Delta E_{ab}$	
Illuminants		Methods	Mean	Max	Mean	Max	Mean	Max
B		PI	0.0343	0.1617	0.9883	0.9997	1.8985	28.3501
		PCA	0.0341	0.1525	0.9886	0.9997	2.6379	22.3945
		Wang [37]	0.0278	0.1048	0.9706	0.9998	3.3742	32.9993
		Zhang [32]	0.0308	0.1409	0.9894	0.9998	1.6845	24.3096
		Proposed	0.0238	0.1067	0.9928	0.9998	0.9643	9.2983
C		PI	0.0350	0.1700	0.9880	0.9997	1.7997	16.1746
		PCA	0.0348	0.1594	0.9883	0.9997	3.6777	29.4821
		Wang [37]	0.0282	0.1149	0.9705	0.9998	2.9507	20.0315
		Zhang [32]	0.0314	0.1509	0.9891	0.9999	1.6168	13.6400
		Proposed	0.0244	0.1142	0.9920	0.9999	1.0146	6.4344
D50		PI	0.0345	0.1636	0.9882	0.9997	2.0576	30.6053
		PCA	0.0343	0.1539	0.9885	0.9997	2.8458	23.8719
		Wang [37]	0.0280	0.1091	0.9705	0.9985	3.4500	36.2491
		Zhang [32]	0.0310	0.1435	0.9893	0.9998	1.8395	26.6381
		Proposed	0.0236	0.1106	0.9927	0.9998	0.9948	8.2185
D65		PI	0.0352	0.1722	0.9879	0.9997	1.9339	8.7401
		PCA	0.0349	0.1614	0.9882	0.9997	3.5937	30.1884
		Wang [37]	0.0284	0.1171	0.9703	0.9998	3.0478	23.8372
		Zhang [32]	0.0316	0.0284	0.1171	0.9998	1.7473	16.0427
		Proposed	0.0248	0.1135	0.9918	0.9999	1.0844	6.2860
E		PI	0.0344	0.1653	0.9882	0.9997	1.6211	18.5733
		PCA	0.0342	0.1554	0.9885	0.9997	3.2294	27.5190
		Wang [37]	0.0267	0.1154	0.9767	0.9998	1.3318	10.2925
		Zhang [32]	0.0310	0.1451	0.9893	0.9998	1.4558	15.5029
		Proposed	0.0237	0.1033	0.9926	0.9998	0.8468	6.0694
F2		PI	0.0375	0.2053	0.9871	0.9997	2.7060	29.6194
		PCA	0.0375	0.1931	0.9872	0.9997	3.1061	19.8124
		Wang [37]	0.0303	0.1526	0.9787	0.9998	2.3323	19.7941
		Zhang [32]	0.0343	0.1857	0.9880	0.9998	2.6457	29.7150
		Proposed	0.0248	0.1135	0.9918	0.9999	1.0844	6.2860
**Average**		PI	0.0352	0.1730	0.9880	0.9997	2.0028	22.0105
		PCA	0.0350	0.1626	0.9882	0.9997	3.1818	25.5447
		Wang [37]	0.0282	0.1190	0.9729	0.9996	2.7478	23.8673
		Zhang [32]	0.0317	0.1324	0.8437	0.9998	1.8316	20.9747
		Proposed	**0.0242**	**0.1103**	**0.9923**	**0.9999**	**0.9982**	**7.0988**

## Data Availability

The Figure 8, Figure 13 and Figure 14 are from University of Eastern Finland, Spectral Color Research Group. Available online: http://www.uef.fi/web/spectral/-spectral-database (accessed on 24 November 2022). Data underlying the results presented in this paper are not publicly available at this time but may be obtained from the authors upon reasonable request.
